# Associations Between Composite Host Vulnerability Score and Transfusion Outcomes After Trauma

**DOI:** 10.3390/medicina62040732

**Published:** 2026-04-12

**Authors:** Yun-Chul Park, Young-Goun Jo, Hyun-Seok Jang, Eui-Sung Jeong, Ji-Hyoun Kang

**Affiliations:** 1 Division of Trauma, Department of Surgery, Chonnam National University Medical School and Hospital, 42 Jebong-ro, Dong-gu, Gwangju 61469, Republic of Korea; 2 Division of Rheumatology, Department of Internal Medicine, Chonnam National University Medical School and Hospital, 42 Jebong-ro, Dong-gu, Gwangju 61469, Republic of Korea

**Keywords:** risk stratification, prognosis, trauma, immune, inflammation

## Abstract

*Background and Objectives*: Outcomes after trauma are traditionally attributed to injury severity and acute physiologic derangement. However, host vulnerability at presentation—reflecting underlying physiologic and nutritional status—may also be associated with bleeding severity and transfusion requirements following acute injury. Whether such vulnerability contributes additional risk information beyond established factors remains incompletely understood. *Materials and Methods*: We conducted a retrospective cohort study of adult trauma patients using a single-center trauma registry. Host vulnerability was assessed using a composite score (CE; range 0–3) based on admission hypoalbuminemia (<3.5 g/dL), anemia (hemoglobin < 11 g/dL), and reduced renal function (estimated glomerular filtration rate < 60 mL/min/1.73 m^2^). Primary outcomes were any blood transfusion and massive transfusion, defined as transfusion of ≥10 units of packed red blood cells within 24 h of admission. Associations between CE score and transfusion outcomes were evaluated using univariable and multivariable logistic regression models adjusted for age, Injury Severity Score (ISS), admission lactate level, and systolic blood pressure (SBP). *Results*: Among 4105 trauma patients, transfusion requirements increased progressively with higher CE scores. Rates of any transfusion rose from 21.7% in patients with CE 0 to 78.6% in those with CE 3, while massive transfusion increased from 1.9% to 23.1% across the same categories. In multivariable analyses, each 1-point increase in CE score was independently associated with higher odds of any transfusion (adjusted odds ratio [aOR] 3.21, 95% confidence interval [CI] 2.80–3.68) and massive transfusion (aOR 1.73, 95% CI 1.45–2.07). *Conclusions*: A composite score reflecting host vulnerability at presentation was associated with bleeding severity and transfusion requirements after trauma, beyond injury severity and acute physiologic factors. These findings suggest that simple laboratory-based markers may provide additional information for early risk stratification of hemorrhagic outcomes after trauma.

## 1. Introduction

Traumatic hemorrhage remains a leading cause of early mortality and morbidity worldwide, accounting for a substantial proportion of potentially preventable deaths after injury. Despite advances in prehospital care, damage control resuscitation, and hemostatic strategies, uncontrolled bleeding continues to drive early physiologic collapse and resource-intensive interventions, including massive transfusion and emergent operative or endovascular procedures. Traditionally, the severity of hemorrhage and transfusion requirements after trauma have been conceptualized primarily as a function of anatomic injury burden and acute physiologic derangement, reflected in established metrics such as the Injury Severity Score (ISS), hypotension, and biochemical markers of shock such as lactate [1,2,3]. While these factors are undeniably central determinants of bleeding severity, they do not fully explain the marked inter-individual variability observed in hemorrhagic outcomes among patients with comparable injury patterns and physiologic presentation.

Growing evidence across acute care disciplines suggests that outcomes following major physiologic stress are strongly influenced not only by the magnitude of the insult, but also by the host’s underlying capacity to respond. Concepts such as physiologic reserve, frailty, and deficit accumulation have been increasingly recognized as modifiers of outcomes in trauma, surgery, and critical illness [4,5,6]. These frameworks emphasize that patients enter acute illness with heterogeneous vulnerability at presentation, shaped by chronic inflammation, nutritional status, organ reserve, and comorbid disease. In trauma populations, pre-injury vulnerability has been linked to higher mortality, complications, and prolonged recovery, even after adjustment for injury severity [7,8,9]. However, the potential role of host vulnerability at presentation in shaping hemorrhagic severity and transfusion requirements—particularly in the early post-injury period—has received comparatively limited attention.

Hemorrhage following trauma is not solely a mechanical consequence of tissue disruption; rather, it reflects a complex interaction between vascular injury, coagulation physiology, inflammatory activation, and metabolic reserve. Acute trauma triggers a systemic inflammatory response characterized by endothelial dysfunction, glycocalyx shedding, platelet activation, and dysregulated coagulation, all of which may amplify bleeding beyond what would be expected from anatomic injury alone [10,11,12]. Importantly, many of these pathways are already perturbed in patients with chronic inflammatory states, malnutrition, anemia, or chronic kidney disease. Such conditions are associated with endothelial fragility, impaired hemostasis, altered platelet function, and diminished capacity to tolerate acute blood loss [13,14,15]. These observations raise the possibility that host vulnerability related to physiologic and nutritional status at presentation may be associated with an increased risk of more severe hemorrhage and higher transfusion requirements after trauma, independent of injury severity.

Among routinely measured laboratory parameters, serum albumin, hemoglobin, and renal function provide integrated signals of chronic inflammation, nutritional reserve, hematologic capacity, and organ dysfunction. Albumin, a negative acute-phase reactant, reflects both systemic inflammation and protein–energy balance; hypoalbuminemia has been consistently associated with increased mortality, complications, and poor outcomes across surgical, medical, and trauma populations [16,17]. Anemia, commonly present in patients with chronic disease, inflammation, or renal dysfunction, limits oxygen-carrying capacity and reduces tolerance to acute blood loss. Admission anemia has been independently associated with increased transfusion requirements and worse outcomes in trauma and surgical cohorts [18]. Reduced renal function, typically operationalized by an estimated glomerular filtration rate (eGFR) < 60 mL/min/1.73 m^2^, reflects diminished organ reserve and is closely linked to chronic inflammation, anemia, platelet dysfunction, and impaired coagulation [19,20]. Together, these parameters capture overlapping but distinct domains of vulnerability at presentation that may influence hemorrhagic response to injury.

Composite indices integrating laboratory markers of inflammation, nutrition, and organ reserve have demonstrated prognostic value in diverse clinical settings. Nutritional and immunologic scores such as the Prognostic Nutritional Index (PNI) and the Controlling Nutritional Status (CONUT) score have been associated with mortality, complications, and transfusion requirements in surgical and cardiovascular populations [21,22]. However, these tools often require multiple variables or were developed outside the trauma context, limiting their applicability in acute trauma workflows and retrospective trauma registries. Furthermore, most trauma-focused studies examining transfusion risk have emphasized injury pattern, shock indices, or resuscitation parameters such as systolic blood pressure, shock index, mechanism of injury, and early hemodynamic markers, with limited exploration of host vulnerability at presentation as a determinant of bleeding severity and transfusion requirements. Existing prediction tools and models predominantly focus on acute physiologic variables and scoring systems designed for early Massive Transfusion Protocol activation rather than pre-injury host vulnerability [23,24].

In recent years, trauma research has increasingly recognized the importance of host factors in modulating response to injury, including susceptibility to coagulopathy, infection, and organ failure [11,25]. Yet, the specific contribution of host vulnerability at presentation to early hemorrhagic outcomes remains incompletely characterized. Understanding whether simple, routinely available laboratory markers can identify patients at heightened risk for severe bleeding and transfusion could have important clinical implications. Early recognition of such vulnerability may inform triage decisions, anticipation of transfusion needs, and tailoring of resuscitation strategies, particularly in resource-limited or time-critical settings.

Accordingly, the objective of this study was to evaluate whether a composite host vulnerability score, derived from admission hypoalbuminemia, anemia, and reduced renal function, is independently associated with bleeding severity and transfusion requirements after trauma. Using a large single-center trauma registry, we examined the relationship between vulnerability at presentation and both any transfusion and massive transfusion, adjusting for established markers of injury severity and acute physiologic derangement. We hypothesized that higher host vulnerability would be associated with greater transfusion requirements, independent of injury burden and shock physiology, thereby highlighting the role of host-related factors in hemorrhagic outcomes following traumatic injury.

## 2. Methods

### 2.1. Study Design and Data Source

We conducted a retrospective cohort study using data from a prospectively maintained trauma registry at Chonnam National University Hospital (CNUH) trauma center. The registry includes detailed demographic, clinical, laboratory, procedural, and outcome data for all trauma patients admitted to the institution. The study period spanned from December 2006 to January 2021 in CNUH. The study protocol was approved by the institutional review board, and the requirement for informed consent was waived due to the retrospective nature of the analysis (CNUH-2026-068).

### 2.2. Study Population

Adult patients (aged ≥18 years) admitted for traumatic injury during the study period were eligible for inclusion. Patients were excluded if key variables required for construction of the composite host vulnerability score—serum albumin, hemoglobin, or estimated glomerular filtration rate—were missing at admission. Patients who died on arrival or had incomplete transfusion records were also excluded from the primary analysis.

### 2.3. Assessment of Host Vulnerability

Host vulnerability at presentation was quantified using a composite score (CE), ranging from 0 to 3, derived from routinely available admission laboratory parameters. One point was assigned for each of the following criteria:

1. Hypoalbuminemia, defined as serum albumin < 3.5 g/dL
2. Anemia, defined as hemoglobin < 11 g/dL
3. Reduced renal function, defined as estimated glomerular filtration rate < 60 mL/min/1.73 m^2^

The CE score was designed to capture a multidimensional vulnerability phenotype reflecting chronic inflammation, impaired nutritional reserve, and reduced organ reserve. These domains are frequently interrelated and commonly observed in patients with chronic inflammatory conditions, multimorbidity, and aging-related physiologic decline. For the construction of the CE score, we used laboratory values obtained at or immediately after hospital admission. Measurements obtained later during hospitalization were excluded to avoid incorporating changes related to ongoing hemorrhage, resuscitation, or clinical deterioration.

### 2.4. Outcomes

The primary outcomes of interest were any blood transfusion and massive transfusion.

Any transfusion was defined as receipt of at least one unit of packed red blood cells during the index hospitalization.

Massive transfusion was defined a priori as transfusion of ≥10 units of packed red blood cells within the first 24 h of admission. Although alternative definitions using shorter time frames have been proposed, we adopted the conventional definition (≥10 units within 24 h) to maintain consistency with prior studies.

### 2.5. Covariates

Baseline demographic variables included age and sex. Injury severity was assessed using the Injury Severity Score (ISS). Acute physiologic derangement was evaluated using admission systolic blood pressure and serum lactate level, measured at presentation. These variables were selected a priori based on their established associations with hemorrhage severity and transfusion requirements in trauma patients and were included as covariates in multivariable analyses.

### 2.6. Statistical Analysis

Baseline characteristics were summarized according to CE score categories and compared using appropriate descriptive statistics. Categorical variables were reported as frequencies and percentages, and continuous variables were reported as medians with interquartile ranges. Associations between CE score and transfusion outcomes were first examined using univariable logistic regression. Multivariable logistic regression models were then constructed to evaluate the independent association between CE score and each transfusion outcome, adjusting for age, ISS, admission lactate level, and systolic blood pressure. The CE score was modeled as an ordinal variable to assess dose–response relationships. Analyses were performed using a complete-case approach, as key variables including lactate, albumin, and eGFR were not available for all patients. Results are reported as odds ratios with corresponding 95% confidence intervals. All statistical analyses were performed using standard statistical software, and a two-sided *p* value < 0.05 was considered statistically significant. Analyses were performed using SPSS ver sion 21.0 (SPSS Inc., Chicago, IL, USA).

## 3. Results

### 3.1. Study Population and Transfusion Outcomes

A total of 4105 adult trauma patients were included in the analysis. Patients were stratified according to the composite host vulnerability score (CE), ranging from 0 to 3. Transfusion-related outcomes across CE categories are summarized in Table 1. Overall, both the frequency and severity of transfusion requirements increased progressively with higher CE scores. Patients with higher CE scores demonstrated substantially greater exposure to blood transfusion during the acute hospitalization period, suggesting a graded relationship between host vulnerability at presentation and bleeding-related outcomes. Of the initial cohort, 2367 patients were included in the complete-case analysis after exclusion of patients with missing data in key laboratory variables. Baseline characteristics of included and excluded patients are compared in Supplementary Materials Table S1.

### 3.2. Association Between CE Score and Any Transfusion

**Figure 1:**
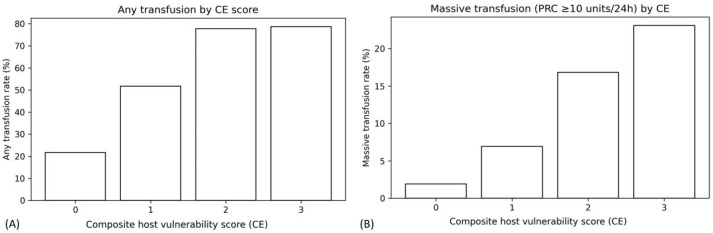
Proportion of patients receiving blood transfusion according to CE score. (**A**) Proportion of patients receiving any blood transfusion according to CE score categories. (**B**) Proportion of patients requiring massive transfusion (≥10 units of packed red blood cells within 24 h of admission) according to CE score categories. In both analyses, the proportion of patients requiring transfusion increased progressively across CE categories, demonstrating a clear dose–response relationship. Error bars represent 95% confidence intervals.

The proportion of patients receiving any blood transfusion increased markedly across CE categories (Figure 1A). Patients with CE 0 had the lowest transfusion rate (21.7%), whereas transfusion was required in more than half of patients with CE 1 (51.7%). This proportion further increased to 77.8% among patients with CE 2 and remained high in those with CE 3 (78.6%). In parallel, the median number of packed red blood cell (PRC) units transfused within the first 24 h increased with CE score. Patients with CE 0 had a median PRC requirement of 0 units, whereas patients with CE 2 and CE 3 required progressively higher transfusion volumes (median 3 and 4 units, respectively), reflecting increasing bleeding severity with greater host vulnerability at presentation (Table 1).

### 3.3. Association Between CE Score and Massive Transfusion

Rates of massive transfusion, defined as transfusion of ≥10 units of PRC within 24 h of admission, also increased stepwise with higher CE scores (Figure 1B). Massive transfusion occurred in only 1.9% of patients with CE 0 but rose to 6.9% in CE 1, 16.8% in CE 2, and 23.1% in CE 3. This clear dose–response pattern indicates that host vulnerability at presentation is associated not only with the likelihood of receiving transfusion but also with the risk of severe hemorrhage requiring large-volume blood replacement.

### 3.4. Association Between CE Score and Transfusion Outcomes

**Figure 2:**
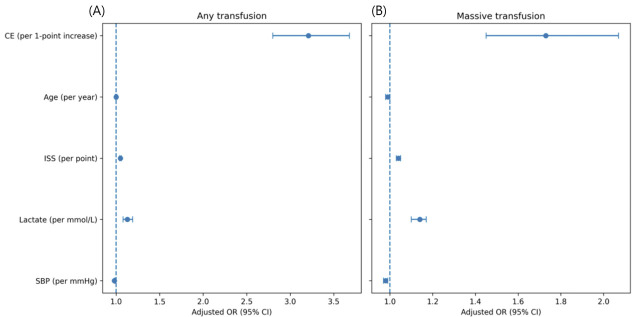
Multivariable-adjusted association between CE score and transfusion outcomes. Forest plots showing adjusted odds ratios (ORs) and 95% confidence intervals for (**A**) any transfusion and (**B**) massive transfusion. Multivariable logistic regression models were adjusted for age, Injury Severity Score (ISS), admission lactate level, and systolic blood pressure. Odds ratios are presented per unit increase for each variable. Higher CE scores were associated with an increased likelihood of both any transfusion and massive transfusion after adjustment for injury severity and physiological parameters.

**Figure 3:**
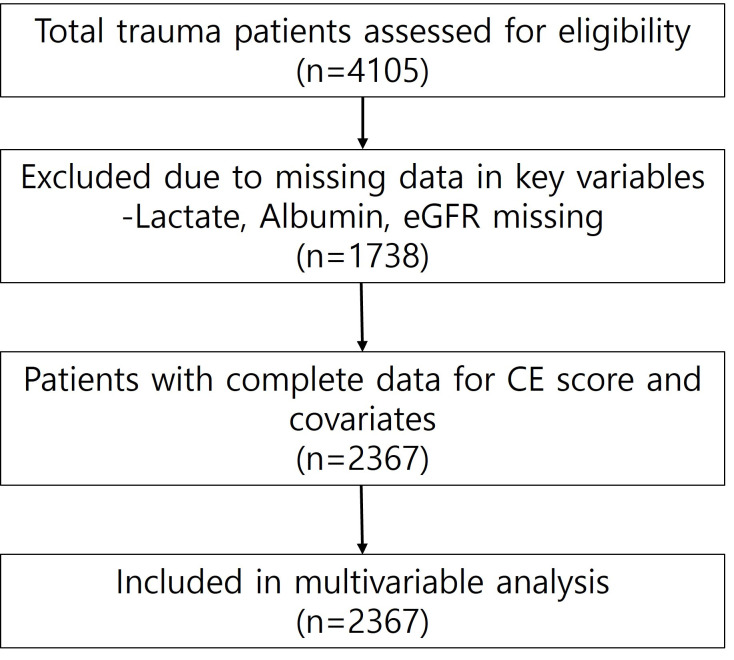
Flow diagram of patient selection. A total of 4105 adult trauma patients were initially assessed. Patients with missing data in key laboratory variables required for CE score construction and multivariable adjustment (lactate, albumin, and eGFR) were excluded. The final analytical cohort included 2367 patients.

In multivariable logistic regression analyses adjusting for age, Injury Severity Score (ISS), admission lactate level, and systolic blood pressure (SBP) at presentation, the CE score remained independently associated with transfusion-related outcomes (Table 2, Figure 2). The selection of patients for multivariable analysis is illustrated in Figure 3. Each 1-point increase in CE score was associated with higher adjusted odds of receiving any blood transfusion during hospitalization (adjusted odds ratio [aOR] 3.21, 95% confidence interval [CI] 2.80–3.68, *p* < 0.001). A similar association was observed for massive transfusion. For each 1-point increase in CE score, the adjusted odds of massive transfusion (defined as transfusion of ≥10 units of packed red blood cells within 24 h of admission) increased by approximately 70% (aOR 1.73, 95% CI 1.45–2.07, *p* < 0.001). Among covariates included in the models, higher ISS and elevated admission lactate levels were independently associated with increased transfusion risk, whereas higher SBP at presentation was associated with a lower likelihood of transfusion. Age was not significantly associated with any transfusion but showed a modest association with massive transfusion. These findings indicate that higher CE scores were consistently associated with increased transfusion requirements after accounting for injury severity and physiological parameters.

### 3.5. Summary of Transfusion-Related Findings

Taken together, these results demonstrate a clear and graded association between the composite host vulnerability score and transfusion-related outcomes after trauma. Higher CE scores were associated with increased likelihood of any transfusion, greater transfusion volume, and higher rates of massive transfusion.

## 4. Discussion

In this retrospective cohort of 4105 adult trauma patients, a simple composite score reflecting admission host vulnerability and physiologic status demonstrated a graded association with hemorrhagic severity as measured by transfusion requirements. Transfusion rates increased markedly across CE strata, and these relationships persisted after adjustment for age, injury severity, and early physiologic derangement. In multivariable models, each 1-point increase in CE score was associated with higher odds of any transfusion and massive transfusion. Collectively, these findings suggest that routinely available admission laboratory markers—capturing vulnerability at presentation rather than injury mechanics alone—may provide additional clinically relevant information for identifying patients at higher risk of transfusion following after trauma.

Most established approaches to predicting hemorrhage and massive transfusion in trauma prioritize injury pattern and acute physiology: hypotension, tachycardia/shock index, FAST positivity, penetrating mechanism, base deficit/lactate, and similar early resuscitation parameters. Scores such as the Assessment of Blood Consumption (ABC) score and the Trauma Associated Severe Hemorrhage (TASH) score were designed to rapidly identify patients likely to require massive transfusion and to trigger activation of massive transfusion protocols (MTP). The ABC score, in particular, is intentionally “bedside-simple,” relying on four non-laboratory variables and has been validated across centers [26,27]. Likewise, the TASH score integrates physiologic and laboratory measures to estimate the probability of life-threatening hemorrhage [28]. These tools have clear operational advantages for time-critical decisions and have meaningfully shaped DCR/MTP workflows. However, even high-performing prediction rules are fundamentally anchored to the acute hemorrhage signal (shock and bleeding already underway). This is appropriate for triggering emergent response, but it leaves a gap: two patients with comparable injury patterns and similar early vital signs can diverge substantially in hemorrhagic trajectory, transfusion needs, and downstream complications. The present findings support the hypothesis that host vulnerability at presentation—captured here by albumin, hemoglobin, and eGFR—contributes additional risk information that is not fully explained by injury severity or a cute physiology. In practical terms, CE may function as a marker of increased host susceptibility that helps explain why some patients decompensate early and require disproportionately high blood product support.

Established transfusion prediction tools, such as the ABC and TASH scores, are primarily designed to identify patients at high risk of massive transfusion based on injury characteristics and early physiologic parameters. These models are well suited for rapid clinical decision-making and activation of massive transfusion protocols. In contrast, the CE score reflects host vulnerability at presentation, capturing aspects of physiologic and organ reserve that are not directly incorporated into existing models. As such, the CE score may provide complementary information, particularly in patients with similar injury severity but differing baseline vulnerability. Rather than replacing established prediction tools, the CE score may be considered as an adjunct for risk stratification, potentially enhancing the identification of patients at risk of severe hemorrhage when used alongside conventional models.

The three CE components each have plausible mechanistic links to hemorrhagic outcomes, and their combination may capture a broader vulnerability construct than any single marker alone. First, hypoalbuminemia is a well-established marker of malnutrition, chronic inflammation, and impaired physiologic reserve, and it has long been associated with adverse outcomes across surgical populations [17]. In trauma, low albumin may reflect pre-existing frailty, hepatic synthetic impairment, chronic disease burden, or inflammatory catabolism—each of which can reduce tolerance to acute blood loss and hemodynamic stress. Albumin also influences oncotic pressure and endothelial integrity; low albumin states are frequently linked to capillary leak and interstitial edema, potentially worsening tissue perfusion and the effectiveness of resuscitation. While albumin itself is not a direct hemostatic factor, hypoalbuminemia plausibly tags patients with poorer compensatory capacity, greater susceptibility to coagulopathy, and increased risk of complications once large-volume resuscitation and transfusion are required [17]. Second, lower hemoglobin at presentation may reduce effective oxygen delivery reserve at the outset of injury. Even modest hemorrhage can precipitate critical oxygen debt in anemic patients, potentially accelerating shock physiology, organ ischemia, and the need for transfusion to restore adequate oxygen-carrying capacity. Trauma cohorts have demonstrated that anemia severity is associated with higher transfusion rates and worse outcomes, supporting the concept that pre-injury hematologic reserve modifies the clinical threshold at which transfusion becomes necessary [29]. Third, reduced eGFR indicates chronic kidney disease (CKD) or impaired renal reserve, which is strongly linked to platelet dysfunction, uremic bleeding tendency, endothelial dysfunction, and systemic inflammation. CKD is also associated with anemia (via reduced erythropoietin), altered coagulation/fibrinolysis balance, and reduced physiologic resilience. Trauma patients with underlying CKD have been reported to experience higher complication rates and mortality after major trauma, consistent with reduced reserve in the setting of acute physiologic stress [30]. These pathophysiologic features provide a coherent rationale for why reduced eGFR on admission may identify patients at higher risk of severe bleeding and transfusion escalation. Importantly, CE is not intended as a direct measure of “immune function” in the narrow immunologic sense (e.g., lymphocyte phenotypes), but rather a pragmatic composite vulnerability construct reflecting host and physiologic status at presentation: chronic inflammation and dysregulated host response, reduced nutritional/hematologic reserve, and impaired organ reserve. This framing aligns with contemporary trauma biology, where early outcomes reflect the interaction between tissue injury/shock and the systemic host response—including trauma-induced coagulopathy, immune dysregulation, and organ failure pathways [25,31].

Trauma-induced coagulopathy (TIC) is increasingly understood as an early, endogenous process triggered by tissue injury and shock, with contributions from endothelial activation, anticoagulant pathways, and hyperfibrinolysis, rather than being solely iatrogenic dilution or consumption [11,25]. While our study did not directly measure viscoelastic profiles or specific coagulation mediators, higher CE scores may be associated with an increased being in or developing a maladaptive hemostatic state—or reduced capacity to compensate for hemostatic disturbances once it develops. Conceptually, the CE score may reflect a combination of pre-existing vulnerability and early physiologic derangement, including endothelial dysfunction and reduced physiologic reserve. This may partly account for differences in clinical trajectories among patients with similar injury burden and early physiologic presentation, including variation in coagulopathy development and transfusion requirements.

From a systems perspective, early recognition of patients at risk for severe bleeding has tangible benefits: proactive MTP activation, preparation of blood products, expedited hemostatic intervention, and optimized resource allocation. Contemporary evidence supports that timely, balanced resuscitation strategies influence early hemorrhagic death and hemostasis achievement, as shown in major observational and randomized studies of transfusion ratios and DCR principles [32,33]. CE offers a complementary axis to existing paradigms: rather than replacing physiology-based triggers, it may help refine risk stratification within borderline cases, retrospective trauma registry analyses, and potentially resource-limited settings where advanced testing is not available.

The practical appeal of CE lies in its simplicity and availability. Albumin, hemoglobin, and creatinine (for eGFR) are commonly obtained on admission and can be abstracted reliably from trauma registries. Unlike some nutritional indices (e.g., PNI or CONUT) that require multiple variables and were largely developed outside trauma workflows, CE focuses on three high-yield markers that are typically present even in retrospective datasets. This may facilitate implementation as (1) a registry-friendly risk adjuster for hemorrhagic outcomes, (2) a bedside adjunct to clinical judgment for early transfusion preparedness, and (3) a screening tool to identify high-risk subgroups for prospective validation or interventional trials.

Several limitations warrant emphasis. First, as an observational retrospective study from a single center, residual confounding is likely despite multivariable adjustment. Important unmeasured factors—including pre-injury anticoagulant and antiplatelet use, chronic liver disease severity, baseline frailty, and prehospital resuscitation details—were not available and may have influenced both the components of the CE score and transfusion outcomes. Second, a substantial proportion of patients were excluded from multivariable analyses due to missing data in key laboratory variables (lactate, albumin, and eGFR). These tests are not routinely obtained in all trauma patients and are more frequently measured in those with greater clinical severity, suggesting that missingness was not random. As a result, the complete-case cohort may have been enriched with more severely ill patients, introducing potential selection bias. Although we compared included and excluded patients and found broadly similar injury severity and mortality, residual bias cannot be excluded. Third, admission laboratory values may reflect not only pre-existing vulnerability but also early physiologic changes related to acute trauma, hemorrhage, hypoperfusion, and resuscitation. Accordingly, the CE score should be interpreted as a marker of vulnerability at presentation rather than a pure measure of baseline status. We were unable to account for prehospital fluid administration, which may have influenced laboratory values at admission. Fourth, transfusion practice reflects both patient condition and clinician or institutional thresholds, and therefore generalizability across centers and protocols requires further validation. Finally, we did not evaluate incremental discrimination or calibration of the CE score relative to established prediction tools (e.g., ABC or TASH) or viscoelastic-guided resuscitation strategies. Future studies are needed to assess the comparative and additive value of the CE score in different clinical settings.

## 5. Conclusions

Our findings suggest that host vulnerability and physiologic status at presentation are associated with hemorrhagic outcomes after trauma. A simple three-variable composite score was associated with substantially higher risk of transfusion and massive transfusion independent of injury severity and early physiologic derangement. If validated externally, the CE score may serve as a pragmatic, registry-compatible adjunct for early hemorrhage risk stratification and may contribute to advancing trauma research beyond injury mechanics alone toward a more complete host–injury interaction framework.

## Figures and Tables

**Table 1:** Transfusion outcomes according to composite host vulnerability score (CE).

CE	N	Any Transfusion Rate, %	Massive Transfusion Rate, %	PRC Units Within 24 h, Median
0	1576	21.7	1.9	0
1	967	51.7	6.9	0
2	1308	77.8	16.8	3
3	234	78.6	23.1	4

Values are presented as number of patients (N), percentage, or median as appropriate. Massive transfusion was defined as transfusion of ≥10 units of packed red blood cells within 24 h of admission.

**Table 2:** Multivariable-adjusted association between CE score and transfusion outcomes.

Variable	Any Transfusion, OR (95% CI)	Any Transfusion, *p*-Value	Massive Transfusion, OR (95% CI)	Massive Transfusion, *p*-Value
CE (per 1-point increase)	3.21 (2.80–3.68)	<0.001	1.73 (1.45–2.07)	<0.001
Age (per year)	1.00 (0.99–1.01)	0.89	0.99 (0.98–1.00)	0.007
ISS (per point)	1.05 (1.04–1.06)	<0.001	1.04 (1.03–1.05)	<0.001
Admission lactate (per mmol/L)	1.13 (1.08–1.19)	<0.001	1.14 (1.10–1.17)	<0.001
Systolic BP (per mmHg)	0.98 (0.98–0.99)	<0.001	0.98 (0.97–0.99)	<0.001

Analytic dataset: complete-case analysis (N = 2367 of 4106 total patients). Odds ratios (ORs) were estimated using multivariable logistic regression models adjusted for age, Injury Severity Score (ISS), admission lactate level, and systolic blood pressure (BP) at presentation. Massive transfusion was defined as transfusion of ≥10 units of packed red blood cells within 24 h of admission.

## Data Availability

Restrictions apply to the availability of these data. Data were obtained from Chonnam National University Hospital and are available (http://www.cnuh.com) with the permission of Chonnam National University Hospital.

## Supplementary Materials

The following supporting information can be downloaded at: https://www.mdpi.com/article/10.3390/medicina62040732/s1, Table S1: Comparison of included and excluded patients.
