# Supplementary material for: Associations Between Composite Host Vulnerability Score and Transfusion Outcomes After Trauma
Source: Medicina (Kaunas). 2026 Apr 12;62(4):732. doi: 10.3390/medicina62040732 (PMC13118004; doi:10.3390/medicina62040732)
Supplement: Supplementary file 1 [file medicina-62-00732-s001.zip › medicina-4213288-supplementary.pdf]

Supplementary Table S1. Comparison of included and excluded patients

Variable	Included (n = 2,367)	Excluded (n = 1,738)	p-value
Age (years)	53.1 [37.1–67.0]	46.1 [29.1–61.0]	<0.001
Male sex, n (%)	1,726 (72.9%)	1,197 (68.9%)	0.108
ISS	17 [10–25]	16 [9–25]	0.123
SBP (mmHg)	100 [80–120]	120 [100–140]	<0.001
Lactate (mmol/L)	2.60 [1.50–4.48]	1.60 [1.00–2.80]	<0.001
Albumin (g/dL)	3.40 [2.80–3.80]	3.90 [3.40–4.30]	<0.001
eGFR (mL/min/1.73m²)	101.6 [84.0–117.1]	109.7 [91.2–123.9]	<0.001
Transfusion, n (%)	1,478 (62.4%)	565 (32.5%)	<0.001
Mortality, n (%)	224 (9.5%)	188 (10.8%)	0.170

Values are presented as median [interquartile range] or number (percentage). Included patients refer to those with complete data for multivariable analysis; excluded patients were omitted due to missing data in key variables. ISS, Injury Severity Score; SBP, systolic blood pressure; eGFR, estimated glomerular filtration rate.
